# *Mycobacterium tuberculosis* Primary Infection and Dissemination: A Critical Role for Alveolar Epithelial Cells

**DOI:** 10.3389/fcimb.2019.00299

**Published:** 2019-08-21

**Authors:** Michelle B. Ryndak, Suman Laal

**Affiliations:** Department of Pathology, New York University School of Medicine, New York, NY, United States

**Keywords:** alveolar epithelial cells, tuberculosis, infection, adhesins, toxins, transcriptome, vaccine

## Abstract

Globally, tuberculosis (TB) has reemerged as a major cause of morbidity and mortality, despite the use of the *Mycobacterium bovis* BCG vaccine and intensive attempts to improve upon BCG or develop new vaccines. Two lacunae in our understanding of the *Mycobacterium tuberculosis* (*M. tb*)-host pathogenesis have mitigated the vaccine efforts; the bacterial-host interaction that enables successful establishment of primary infection and the correlates of protection against TB. The vast majority of vaccine efforts are based on the premise that cell-mediated immunity (CMI) is the predominating mode of protection against TB. However, studies in animal models and in humans demonstrate that post-infection, a period of several weeks precedes the initiation of CMI during which the few inhaled bacteria replicate dramatically and disseminate systemically. The “Trojan Horse” mechanism, wherein *M. tb* is phagocytosed and transported across the alveolar barrier by infected alveolar macrophages has been long postulated as the sole, primary *M. tb*:host interaction. In the current review, we present evidence from our studies of transcriptional profiles of *M. tb* in sputum as it emerges from infectious patients where the bacteria are in a quiescent state, to its adaptations in alveolar epithelial cells where the bacteria transform to a highly replicative and invasive phenotype, to its maintenance of the invasive phenotype in whole blood to the downregulation of invasiveness upon infection of epithelial cells at an extrapulmonary site. Evidence for this alternative mode of infection and dissemination during primary infection is supported by *in vivo, in vitro* cell-based, and transcriptional studies from multiple investigators in recent years. The proposed alternative mechanism of primary infection and dissemination across the alveolar barrier parallels our understanding of infection and dissemination of other Gram-positive pathogens across their relevant mucosal barriers in that barrier-specific adhesins, toxins, and enzymes synergize to facilitate systemic establishment of infection prior to the emergence of CMI. Further exploration of this *M. tb*:non-phagocytic cell interaction can provide alternative approaches to vaccine design to prevent infection with *M. tb* and not only decrease clinical disease but also decrease the overwhelming reservoir of latent TB infection.

## Introduction

Tuberculosis (TB) is emerging as the most important infectious disease of our times, and is the leading cause of morbidity and mortality due to any infectious disease worldwide, surpassing even HIV. In 2015, there were >10 × 10^6^ cases of TB and >1 × 10^6^ TB-related deaths (Murray and Collaborators, 2018). Estimates are that ~ 2 × 10^9^ people, a third of the global population carries a latent TB infection (LTBI). Approximately 10% of these infections will reactivate to progress to infectious clinical TB in their lifetime, thus maintaining transmission in humans. The risk for TB, both primary and reactivated, is higher in immune-compromised individuals; at least a third of HIV-related deaths are attributed to TB.

The current vaccine, *Mycobacterium bovis* bacilli Calmette-Guerin (BCG), developed in the 1920's, is effective in attenuating severe disseminated forms of TB in children, but not in preventing primary infection or reactivation of LTBI in adults. To improve the efficacy of BCG, approaches such as development of recombinant subunit vaccines, and engineering BCG to improve protection by inserting genes for *Mycobacterium tuberculosis* (*M. tb*) antigens, mammalian cytokines, host resistance factors, bacterial toxin-derived adjuvants etc. have been used (Nieuwenhuizen and Kaufmann, 2018). The goal of the vaccine candidates is to induce robust immune responses critical for controlling progression of *M. tb* infection. Candidate selection is based primarily on their ability to elicit IFN-γ from T cells *in vitro* and reduce bacterial burden in animal models *in vivo*. Unfortunately, the correlates of protection against TB are not completely defined, and the immune responses elicited by the candidate vaccines selected so far have failed to correlate with protection in humans.

The most successful anti-TB immune response in humans is the adaptive immunity elicited by natural infection, which prevents ~90–95% of infected individuals from developing active TB. This immune response prevents both progression of primary infection to clinical TB and reactivation of LTBI for long durations. So far, no candidate vaccine has achieved this. Importantly, the naturally elicited adaptive immune responses (a.k.a. cell-mediated immunity, CMI) that successfully maintain latency for the lifetime of ~90% of the infected individuals, fail to eradicate the existing infection and cannot protect against subsequent infections with *M. tb* (Barrios-Payán et al., 2012; Laal, 2012).

Multiple investigations of TB household-contacts performed for the identification of undiagnosed/sub-clinical cases (Beyanga et al., 2018; Fox et al., 2018; Ohene et al., 2018) as well as to specify the risk factors that influence an exposed person to active disease have been performed (Jones-Lopez et al., 2017; Stein et al., 2018). Meta-analysis of these studies demonstrates that, as measured by conversion to positive tuberculin skin test (TST), despite similar exposure, *M. tb* infection is established in only ~50% of the contacts (Morrison et al., 2008). Moreover, studies on transmission of TB in households indicate that <20% of TB transmission occurs via household contact, demonstrating that frequent close encounters do not necessarily result in infection (latent or active) (Martinez et al., 2017). It is unclear how contacts who do not convert to TST positivity despite frequent exposure to *M. tb* prevent the establishment of infection, although studies of genetic susceptibility suggest that TNF-mediated effector mechanisms may influence innate resistance to *M. tb* infection (Abel et al., 2018).

The precise events that occur during primary infection are poorly understood. There are no external signs and symptoms of TB infection, and it can take up to 8 weeks post-infection (p.i.) for TST reactivity to become positive and for TB-specific IFN-γ producing cells to appear (CDC, 2013). The events that occur in the lungs prior to the onset of these immune responses remain unexplored in humans where neither the time of infection nor the inhaled dose can be ascertained. In animal models, both the time and dose of infection can be controlled, but the paucity of bacterial numbers inhaled and the large pulmonary tissue volume are problematic. Yet this “Black Box” is where the dynamic events that determine the subsequent course of *M. tb* infection occur.

In this review, we discuss the current understanding of primary infection with *M. tb* and the missing information regarding the pre-CMI events that lead to establishment of infection. We discuss the features of the alveolar barrier, describe the potential mechanisms for *M. tb* dissemination across this barrier, and demonstrate that these are parallel to mechanisms used by other bacterial pathogens to cross their pertinent physiological barriers. Using the published transcriptional profiles of *M. tb* in environments relevant to those encountered during the establishment of infection, we have synthesized a narrative of how the inhaled *M. tb* adapts to and/or exploits each step in its infection and dissemination journey to its benefit. The focus of this review is the potentially critical role of the alveolar epithelial cell (AEC) both as a permissive niche for *M. tb* replication and as a portal for systemic *M. tb* dissemination. These interactions that occur during primary infection can be targeted in novel vaccine strategies to prevent the establishment of *M. tb* infection.

## Current Understanding of Primary Infection

Only a subset of the bacteria-laden aerosol droplets (<5 μm diameter with 1–3 bacilli) inhaled actually reach any alveolar sac; the larger size droplets (>5 μm) are trapped in the upper respiratory system by mucus and ciliary action (Fernández Tena and Casan Clarà, 2012). Current understanding is that the inhaled bacteria are phagocytosed by alveolar macrophages (AM). Several mechanisms that *M. tb* employ to subvert being killed and to replicate intracellularly have been described, including inhibition of phagosomal maturation, de-acidification of the phagosomal vacuole, escape from the vacuole to the cytosol, and modulation of macrophage apoptosis (Awuh and Flo, 2016); understandably, almost all these studies have not been performed in AM. In any case, infected macrophages recruit additional circulating monocytes/macrophages and neutrophils to the site of infection via expression of chemokines (CCL2; CXCL10; and TNF) (Jang et al., 2008; Deshmane et al., 2009; Domingo-Gonzalez et al., 2016); and the newly recruited cells in turn phagocytose the bacteria released by lysis of the infected AM. Recent investigations of primary infection in the mouse high-dose aerosol infection model show that AMs are more permissive for *M. tb* replication than interstitial macrophages (Huang et al., 2018), and that *M. tb*-infected AM migrate to the lung interstitium where they can be visualized as small aggregates at ~2 weeks p.i. (Cohen et al., 2018); these early cellular foci are postulated to be the precursors to lung granulomas. By ~3 weeks p.i., *M. tb* are present in recruited monocytes and neutrophils in greater numbers than the initially infected AM (Cohen et al., 2018). The bacteria-laden phagocytic cells transport their cargo via diapedesis across the alveolar barrier to lymph nodes (“Trojan Horse” mechanism) (Nguyen and Pieters, 2005; Wolf et al., 2008) where CMI responses are initiated around 6–8 weeks p.i. in humans (Wallgren, 1948; CDC, 2013). This time point is on the early boundary of CMI and contributes to the containment of *M. tb* infection as measured by bacterial burden, disease progression, and ability to prevent the reactivation of LTBI (Chen et al., 2009; Lin et al., 2012).

## Events Preceding Initiation of CMI

During the first weeks of aerosol infection, dramatic bacterial replication (>20,000-fold) is reported to occur in the lung (Wolf et al., 2008). Older literature reported that *M. tb* spreads from the site of inoculation to multiple organs within days (Soltys and Jennings, 1950). Additional support for *M. tb* replication and systemic dissemination from the lungs prior to elicitation of CMI also comes from other studies in animal models. Thus, in aerosol-infected mice, *M. tb* is detected in the spleens and livers as early as 2 weeks p.i. (Chackerian et al., 2002), and bacteria are detected in spleens of guinea pigs within 3 weeks after a low-dose (3 CFU) aerosol-infection (Mcmurray, 2003). Tubercle lesions with granulomatous inflammation and necrosis appear in the lymph-nodes, spleen, liver, pancreas, adrenal glands and heart at ~4 weeks post—low-dose aerosol-infection with virulent strains of *M. tb* (Erdman K01, CDC1551, and HN878) (Palanisamy et al., 2008). In humans, evidence for systemic dissemination prior to elicitation of CMI comes from studies of autopsy tissues from individuals who died of unrelated causes in a TB-endemic setting. In these individuals, LTBI was demonstrated in the liver, kidneys, and spleen in the absence of tubercular lesions (Barrios-Payán et al., 2012). Importantly, the extrapulmonary (EP) LTBIs are in multiple non-phagocytic cell types such as Bowman's capsule parietal cells and convoluted proximal tubule epithelial cells in the kidneys, sinusoidal EC and Kupffer cells in the spleen, and hepatocytes and portal biliary duct epithelial cells in the liver. These studies demonstrate the pre-CMI systemic dissemination of *M. tb* in animals and humans and also highlight the breadth of cell types that *M. tb* infects and survives in *in vivo* (Barrios-Payán et al., 2012).

## Breaching the Alveolar Barrier to Disseminate Systemically

**Figure 1:**
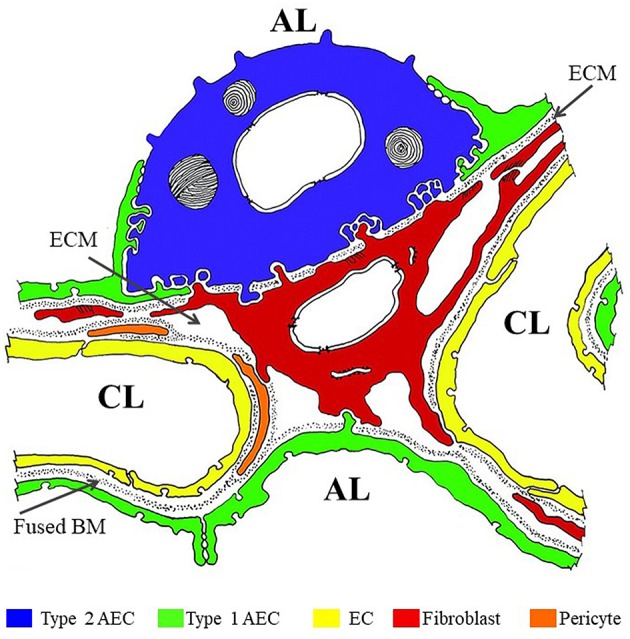
Color overlay of trans-electron micrograph of the alveolar barrier. Adapted from Burns et al. (2003) with author's permission (Dr. Alan R. Burns) and the permission of Physiological Reviews (American Physiological Society). AL, alveolar lumen; CL, capillary lumen; ECM, extracellular matrix; BM, basement membrane. Blue = Type 2 AEC; Green = Type 1 AEC; Yellow = EC; Red = fibroblast; Orange = pericyte.

The alveolar barrier is the critical obstacle the inhaled *M. tb* must traverse to disseminate systemically (Figure 1) (Burns et al., 2003). The alveolar lumen is lined by type 1 and type 2 AECs which rest on a basement membrane (BM) composed primarily of extracellular matrix (ECM) comprised of laminin (Lm), collagen, entactin, heparan sulfate proteoglycans (HSPGs), and other additional minor components. Alveolar sacs are wrapped with alveolar capillaries lined with endothelial cells (EC) also resting on their own BM. The BMs underlying the AEC and EC are fused together in areas where gas exchange occurs (thin wall); in areas not involved in gas exchange, the two BMs are separated by ECM interspersed with interstitial fibroblasts, pericytes etc. (thick wall). The lumen of an average alveolus has ~28,000 thin and large flat type 1 AECs that cover 90–95% of the surface area (Schneeberger, 1991). The primary function of these cells is to facilitate gas exchange. About 5–10% of the alveolar lumen is occupied by ~1,500–2,000 cuboidal type 2 AEC that are responsible for secreting surfactants and restoring damaged type 1 AEC (Schneeberger, 1991). In addition, ~50 AM surveil the alveolus for foreign particles (Crystal, 1991). The lack of a type 1 AEC line has precluded studies of the host-pathogen interaction with these cells. In contrast, *M. tb* infection of and replication in A549 cells, a human type 2 AEC carcinoma cell-line commonly used as a model for the study of pulmonary diseases, is well-investigated (Mcdonough Kathleen and Kress, 1995; Bermudez and Goodman, 1996, Birkness et al., 1999; Dobos et al., 2000; Bermudez et al., 2002; Castro-Garza et al., 2002; Garcia-Perez et al., 2003; Chapeton-Montes et al., 2008; Ryndak et al., 2015). A549 cells maintain the type 2 AEC characteristics such as multilamellar bodies containing phospholipids similar to primary type 2 AEC and have similar surfactant protein expression (Nardone and Andrews, 1979; Cooper et al., 2016). However, while A549 cells have served as a useful type 2 AEC model across varied pulmonary disease studies (e.g., Gonzalez-Juarbe et al., 2017; Laventie et al., 2019; Nachmias et al., 2019; Nickol et al., 2019), and are capable of self-renewal, this cell line is not capable of differentiating to type 1 AEC, and therefore, stimuli that would trigger type 1 differentiation *in vivo* would go unnoticed (Fuchs et al., 2003). We have earlier demonstrated that lung fibroblasts (WI-26 cells) are exquisitely susceptible to *M. tb*-induced lysis; whether they also support *M. tb* replication is not known (Kinhikar et al., 2010). It may be speculated that, like pulmonary fibroblasts, type 1 AEC may also be very sensitive to *M. tb*-mediated lysis and could therefore present a site for enhanced traversal across the alveolar barrier.

The accepted mechanism for *M. tb* dissemination across the alveolar barrier is that AM phagocytose the inhaled bacteria then traverse the barrier via diapedesis, a.k.a. the “Trojan Horse” mechanism (Nguyen and Pieters, 2005). In the last decade or so, there has been steady accumulation of evidence for a potential role for AEC in primary infection that leads us to propose an additional scenario on events that occur during this “Black Box” of primary infection. Based on our studies and those by multiple investigators discussed here, we propose that, in addition to the “Trojan Horse” mechanism, dramatic replication of *M. tb* in type 2 AEC and direct migration of free bacteria across the alveolar barrier into the circulation contributes significantly to the systemic dissemination from the lung during primary infection.

**Figure 2:**
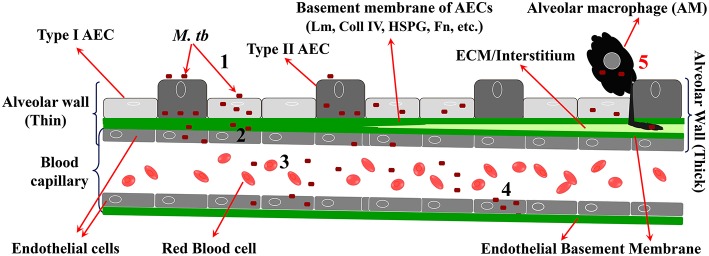
Steps in migration of *M. tb* across the alveolar barrier. 1 = *M. tb* adheres to, invades, and replicates in AEC; 2 = *M. tb* penetrates the BM and EC; 3 = *M. tb* exits the EC to enter the circulation; 4 = *M. tb* infects EP EC to establish LTBI in EP sites. 5 = “Trojan Horse” mechanism by which *M. tb* crosses the barrier via infected AM.

To cross the alveolar barrier directly, the few inhaled *M. tb* would have to invade AEC, replicate intracellularly, damage the BM, invade EC (and perhaps replicate in them), and then exit to the capillary lumen (Figure 2). This is in line with recent studies on mycobacterial penetration of the blood:brain barrier where *Mycobacterium marinum* has been demonstrated to transmigrate both via infected-macrophages and by a macrophage-independent mechanism (van Leeuwen et al., 2018). Evidence for *M. tb* infection of type 2 AEC has been demonstrated in humans (Hernandez-Pando et al., 2000; Eum et al., 2010; Barrios-Payán et al., 2012). Indirect evidence for massive *M. tb* replication in a macrophage-independent host cell in the lungs comes from studies in aerosol-infected mice that reported >20,000-fold replication at 2 weeks p.i. and prior to CMI in host cells that are “non-migrating and non-antigen presenting” (Wolf et al., 2008). While *M. tb*-uptake by A549 cells is less efficient compared to professional phagocytes, once inside, *M. tb* replicates ~15–20-fold more in the former cells over the same period of time (Mehta et al., 1996). Mechanisms by which type 2 AEC can directly internalize *M. tb* have been reported (Scordo et al., 2016), and some discussion on the potential mechanisms for *M. tb* uptake by AEC is provided later in the current review. Virulent strains of *M. tb* (*M. tb* H37Rv, Erdman, and CDC1551) have been shown to cause permeation and necrosis (cytotoxicity) and cell-cell bacterial spread in A549 monolayers (Mcdonough Kathleen and Kress, 1995; Dobos et al., 2000), while *M. bovis* BCG is attenuated for this phenotype (Dobos et al., 2000). *M. tb*-mediated cytolytic activity is critically associated with virulence, and lysis of the alveolar epithelium could have a substantial effect on downstream *M. tb* dissemination (Hsu et al., 2003; Lewis et al., 2003).

*M. tb* DNA has also been detected in both lung EC and fibroblasts of subjects with LTBI (Hernandez-Pando et al., 2000; Barrios-Payán et al., 2012). EC are reported to be relatively resistant to infection by *in vitro*-grown *M. tb*; however, bacteria that have replicated inside A549 cells are reported to be far more invasive for EC (>90-fold at 24 h p.i.) (Bermudez et al., 2002). Together, these studies indicate that during replication in AEC, *M. tb* acquires increased invasiveness for other non-phagocytic cells.

## ESAT- 6 participates in traversal of free *M. tb* Across the alveolar barrier

The strong memory B and T cell response to ESAT-6 by production of IFN-γ in individuals with LTBI is indicative of its *in vivo* expression during primary infection (Sebina et al., 2014; Pathakumari et al., 2017). Comparative studies identified 16 regions of difference (RD1-16) between the genomes of *M. tb* and BCG, of which one deletion, termed “RD1,” is absent from all BCG sub-strains used as TB vaccines globally. RD1 is part of a 15-gene locus (ESX-1), which encodes a type 7 secretion system (T7SS) that enables the secretion of several proteins including ESAT-6 and CFP10, which are also encoded in RD1. *M. bovis* BCG, *M. tb* ΔRD1 and *M. tb* Δ*esat-6* mutants are attenuated for cytolysis of type 2 AEC and macrophages, cell-to-cell spread, pulmonary necrosis, and bacterial dissemination from the lungs *in vivo* (Mcdonough Kathleen and Kress, 1995; Dobos et al., 2000; Hsu et al., 2003; Lewis et al., 2003; Guinn et al., 2004; Kinhikar et al., 2010). *M. tb* ΔRD1 is also attenuated for lethality in both SCID and BALB/c mice (Hsu et al., 2003), thus correlating the cytotoxic effect with virulence. ESAT-6 can lyse artificial lipid bilayers (Hsu et al., 2003), liposomes (de Jonge et al., 2007), RBCs (Smith et al., 2008), macrophage phagosomes (Simeone et al., 2012), fibroblasts (Tsai et al., 2009), lung fibroblasts and AEC (Kinhikar et al., 2010). Clearly, ESAT-6-mediated lysis is not restricted to phagosomal membranes. Support for ESAT-6 participation in the dissemination of free *M. tb* across the alveolar barrier also comes from studies with *in vitro* bilayer models comprised of monolayers of A549 cells and EC with or without a BM between them (Bermudez et al., 2002; Ryndak et al., 2016). The apical AEC side of these models represents the alveolar lumen, and the basolateral EC side represents the alveolar capillary lumen (entrance to the circulation). In both *in vitro* models, *M. tb* migrates across the barrier in the absence of macrophages, and BCG is attenuated for this ability. Furthermore, in the model containing a BM, in addition to BCG, *M. tb* Δ*esat-6* and *M. tb* Δ*phoP* (impaired for ESAT-6 expression and secretion, respectively) are similarly attenuated for macrophage-free transmigration (Ryndak et al., 2016). These studies collectively demonstrate that ESAT-6 secretion is an important participant in dissemination of *M. tb* from the lungs. Interestingly, the *M. marinum* macrophage-independent migration across the blood:brain barrier is also ESX-1-dependent (van Leeuwen et al., 2018), and therefore, the mechanism proposed below is not unprecedented for mycobacterial dissemination across physiological barriers.

## *M. tb* Transcriptional Adaptations Provide Insight into Primary *M. tb* Infection

Transcriptional analysis has provided insight into how *M. tb* adapts in hostile environments such as the hypoxia and starvation encountered in the granuloma (Betts et al., 2002; Gupta and Chatterji, 2005; Hudock et al., 2017), and in macrophages (Schnappinger et al., 2003; Fontán et al., 2008). Transcriptome analysis of *M. tb* infecting mice lungs has also described a three-stage adaptive process from acute infection (aerobic, replicating) to a transitional phase [transition to nitrate respiration, induction of DevR (DosR) regulon] to a dormant state (non-replicative, nitrate respiration) (Shi et al., 2005). Thus, transcriptional analysis provides discrimination of active and latent TB primarily by aerobic vs. alternative pathways of respiration, active vs. non-replication, and suppression vs. induction of the DevR (DosR) regulon.

To gain insight into primary infection by *M. tb* and its dissemination, we have examined the transcriptional states of *M. tb* in expectorated sputum from smear positive TB patients (Sharma et al., 2017), bacteria replicating in A549 cells (Ryndak et al., 2015), bacteria replicating *ex vivo* in whole blood both from HIV- and HIV+ individuals (Ryndak et al., 2014), and since earlier studies showed that the disseminated bacteria reside in non-phagocytic cells in EP sites, *M. tb* in a human retinal pigment epithelial cell (RPE) line (Rao et al., 2006; Barrios-Payán et al., 2012; Abhishek et al., 2018) (Table 1). The RPE cell-line has earlier been demonstrated to be an accurate functional model for studies of RPE and ocular TB (Ablonczy et al., 2011; La Distia Nora et al., 2018).

**Table 1:** Summary of *M. tb* transcriptional profiles in physiologically relevant environments encountered during establishment of infection.

**Association**	**Gene category**	**Sputum (a)**	**AEC (b)**	**HIV-blood (c)**	**HIV+blood (c)**	**Act mac (d)**	**RPE (e)**
Active replication	30S (23)	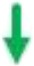 (6)	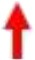 (5)	NDE	NDE	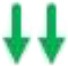 (13)	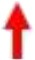 (3)
	50S (36)	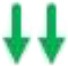 (16)	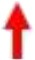 (5)	NDE	NDE	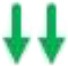 (16)	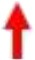 (4) 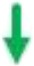 (1)
	ATP Synthase (8)	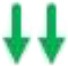 (7)	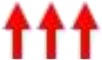 (8)	NDE	NDE	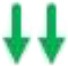 (7)	NDE
Aerobic respiration	NADH-D1 (14)	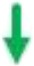 (6)	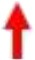 (7)	NDE	NDE	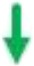 (6)	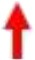 (3) 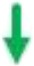 (2)
	Cytochrome C Reductase (3)	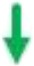 (2)	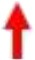 (2)	NDE	NDE	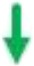 (2)	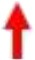 (1) 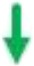 (1)
	Cytochrome C Oxidase	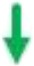 (1)	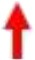 (1)	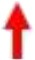 (1)	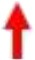 (1)	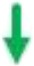 (2)	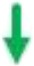 (1)
Dormancy/ Stress	DosR Regulon (48)	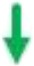 (6)	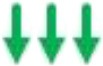 (26)	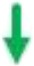 (3)	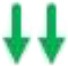 (21)	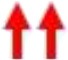 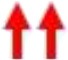 (41)	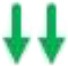 (13)
	Universal Stress Proteins (9)	NDE	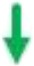 (5)	NDE	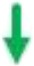 (4)	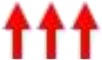 (7)	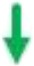 (4)
Resuscitation	Resuscitation Promoting Factors (5)	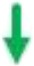 (2)	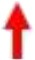 (2)	NDE	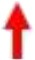 (1)	NDE	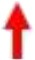 (1)
Persistence	*mprA* (Rv0981)	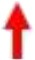	NDE	NDE	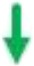	NDE	NDE
Virulence/ ESAT-6 Secretion	ESX-1 (20)	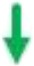 (5)	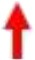 (1)	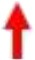 (4)	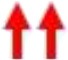 (6)	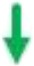 (2)	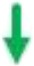 (3) 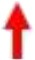 (1)
Iron acquisition	ESX-3 (11)	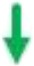 (5)	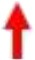 (6)	NDE	NDE	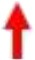 (3)	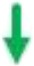 (2)
Virulence/PE/ PPE Secretion	ESX-5 (17)	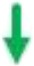 (4)	NDE	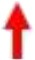 (1)	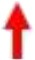 (5)	NDE	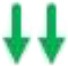 (6) 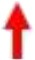 (1)
Virulence/ Dissemination	esat-6 (Rv3875)	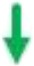	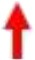	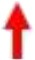	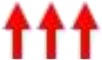	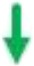	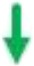
Unknown	Encodes ESAT-6-like Proteins (23)	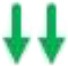 (8)	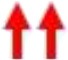 (6)	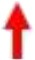 (2)	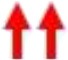 (8)	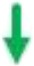 (1)	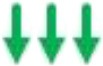 (14)

*Green (down) arrows indicate down-regulation. Red (up) arrows indicate upregulation. Numbers of arrows indicate the extensiveness of differential expression. Numbers in parentheses indicate number of genes within the category or the number of genes differentially expressed. NDE, not differentially expressed. Reference in all studies was M. tb logarithmically-growing in laboratory media. Studies cited (a) (Sharma et al., 2017) (b) (Ryndak et al., 2015) (c) (Ryndak et al., 2014) (d) (Schnappinger et al., 2003) (e) (Abhishek et al., 2018). (AEC, human type 2 AEC, A549 cell line; Act Mac, activated murine bone marrow derived macrophages; RPE, human retinal pigmented epithelial cells, ARPE-19 cell line)*.

The transcriptome of sputum-derived *M. tb* revealed a low energy, low replicating, non-aerobic, inert state (Sharma et al., 2017). These results are similar to transcriptional studies which also demonstrated that *M. tb* in patient sputum is in a persistent, non-replicating state (Garton et al., 2008). Other studies with sputum, broncho-alveolar lavage (BAL) fluid, and cavities from TB patients show that the most abundant cells in the sputum and the BAL are heavily bacteria-laden neutrophils (Eum et al., 2010). This intra-neutrophil localization of the sputum bacteria and their inert state suggests that after exiting the caseum of the cavities, neutrophils transport the bacilli from lesions to the pulmonary airway whereby *M. tb* undergoes a final adaptation, which enables it to survive in these cells as well as the external environments that may be encountered post-expectoration. Interestingly, *M. tb* in the sputum upregulate *mprA*, which encodes a “persistence”-related two-component system response regulator, but not the DevR (DosR) regulon, a hallmark of transition to dormancy/latency. The sputum *M. tb* also down-regulate *esat-6*, and genes involved in the synthesis of virulence-associated cell wall components, PDIM/PGLs. We interpret these cumulative observations to indicate a conservation of energy and weaponry not needed until actual entry into the new host and initiation of infection occurs.

When these metabolically inert bacteria are inhaled into the alveolus, they could be phagocytosed by AM and/or taken up by AEC. The transcriptome of *M. tb* in AM is not defined, but in THP-1 cells (Fontán et al., 2008) and in naïve and INF-γ activated murine bone marrow derived macrophages (mBMM) (Schnappinger et al., 2003), the *M. tb* transcriptome indicates suppression of replication and aerobic respiration, and dramatic upregulation of stress responses associated with transition to dormancy. Thus, genes for 30 and 50S ribosomal protein subunits (translational machinery) and ATP synthase (energy) are down-regulated, and genes for Universal Stress Proteins (response to hypoxia), starvation-induced genes, and the DevR (DosR) regulon (survival during hypoxia and NO stress reactive dormancy) are upregulated. The transcriptome of *M. tb* incubated with the lysosomal soluble fraction from activated mBMM further confirms the non-replicative and stressed state of *M. tb* in the intra-activated-macrophage environment (Lin et al., 2016). Transcripts for *esat-6* were either not differentially expressed (in THP-1) (Fontán et al., 2008) or were down-regulated (in activated mBMM) (Schnappinger et al., 2003). Importantly, *M. tb* infection of primary human AM (hAM) causes them to undergo a metabolic shift to glycolysis leading to increased levels of pro-inflammatory IL-1β and decreased production of the anti-inflammatory IL-10, resulting in enhanced intracellular bacterial killing which could contribute to early clearing/control of *M. tb* infection in humans (Gleeson et al., 2016). Therefore, the AM may not be the most hospitable environment to promote *M. tb* infection.

In stark contrast, the transcriptome of *M. tb* in type 2 AEC offers a scenario in which *M. tb* perceives an environment permissive for replication, an active metabolic state, and increased virulence (Ryndak et al., 2015). Thus, indicators of enhanced replication (genes for 30 and 50S ribosomal protein subunits, ATP synthase), cell wall remodeling (mycolic acid synthesis), aerobic respiration (genes for NADH-DH1, cytochrome c reductase, and cytochrome c oxidase) and virulence (*esat-6*) are upregulated. Furthermore, in contrast to in macrophages, in AEC, genes involved in alternative electron transfer and non-aerobic respiration, and genes encoding Universal Stress Proteins and other hypoxia-induced genes such as those of the DevR (DosR) regulon are down-regulated indicating a perception of reduced stress by the intracellular *M. tb*. Conversely, genes encoding “resuscitation promoting factors” implicated in reactivation from dormancy are upregulated. It is possible that these may also promote the “resuscitation” of the “inert” bacteria expectorated from infectious individuals upon reaching an alveolus in a new host. Another striking difference between the transcriptome of *M. tb* replicating in AEC vs. macrophages is the upregulation of not only *esat-6* but also 5 genes encoding ESAT-6-like proteins; EsxH, EsxJ, EsxK, EsxP, and EsxW, in the former cells. The ESAT-6-like family of proteins is characterized by their small size (~100 amino acids), helix-turn-helix structure, central WXG motif, and flexible N- and C-termini (Pallen, 2002). For ESAT-6, the termini serve as anchors and the helices insert to create the membrane-spanning pore (Ma et al., 2015). Since bacterial pore-forming toxins often belong to families of shared structure (Gouaux et al., 1997; Jedrzejas, 2001; Gilbert, 2010; Heuck et al., 2010), it is possible that, in addition to ESAT-6, other ESAT-6-like family members may also have toxin/pore-forming capabilities, although this remains to be investigated.

Thus, bacteria that have replicated in AEC acquire a virulent phenotype and could traverse the alveolar barrier to enter the circulation for dissemination. While *M. tb*-uptake by EC is enhanced after passage through AEC, it is not known if *M. tb* replicates further in EC prior to entering the circulation. The transcriptome of *M. tb* replicating in whole human blood *ex vivo* mirrors that of *M. tb* in AEC in suppression of dormancy (down-regulation of genes within the DevR (DosR) regulon), support for cell wall remodeling (upregulation of genes involved in PDIM/PGL synthesis) and enhanced virulence (upregulation of genes within the ESX-1 locus; including *esat-6*) (Ryndak et al., 2014). Importantly, these adaptations are more dramatic in whole blood from HIV+ patients, both in numbers of differentially expressed *M. tb* genes and the degree to which they are differentially expressed. Thus, while 3/48 genes from the DevR (DosR) regulon are downregulated in the HIV- blood, 21/48 are downregulated in the HIV+ blood. Also, 3 genes involved in PDIM/PGL synthesis are upregulated by *M. tb* in the HIV- blood while 10 are up in the HIV+ blood. Furthermore, while 4 genes of the ESX-1 locus are upregulated by *M. tb* in HIV- blood (including *esat-6*), 6 genes are upregulated in the HIV+ patient blood. *esat-6* is the most upregulated *M. tb* gene in the HIV+ blood, and 7 genes encoding ESAT-6-like proteins that are not differentially expressed in the HIV- blood, are upregulated in the HIV+ blood (The function of these ESAT-6-like proteins is not yet known). It is after this hematogenous spread that *M. tb* would enter the non-phagocytic cells in multiple EP sites.

In contrast to the transcriptome in AEC where *M. tb* acquires a virulent and invasive phenotype and in blood where this phenotype is maintained, the *M. tb* transcriptome in RPE cells is that of a “quiet” but not latent state (Abhishek et al., 2018). Thus, while many stress-related genes are downregulated in RPE, these cells are not dramatically permissive for *M. tb*. While a small number of genes encoding the translational machinery (50 and 30S ribosomal subunits) are upregulated, none of the genes encoding ATP synthase are differentially expressed (compared to 8/8 upregulated in AEC). Several genes within the virulence-associated ESX-1 and ESX-5 T7SS loci are downregulated including *esat-6*, and the 5 genes encoding ESAT-6-like proteins that are upregulated in both AEC and HIV+ blood, plus an additional 9 in this family, are downregulated. This pattern of transcription indicates that while in AEC the drive is toward dramatic replication, virulence, and dissemination, and the potential for virulence and dissemination is maintained in the blood, but in the EP sites, the goal may be to lie quietly and avoid detection by the immune responses elicited. Detection of LTBI is an important component of intraocular TB diagnostic criteria since it often occurs in the absence of active pulmonary TB or other forms of EPTB. This indicates that the bacteria likely disseminated to the ocular site during primary infection.

**Figure 3:**
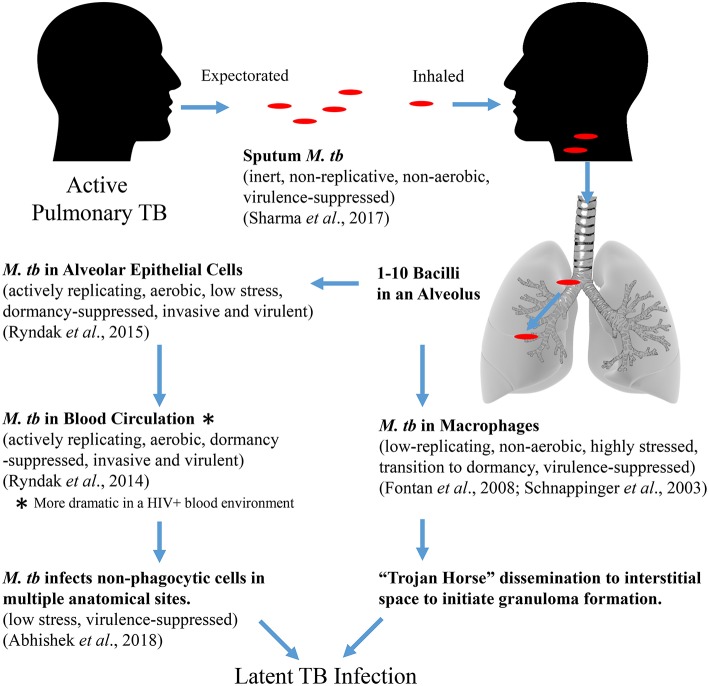
Proposed *M. tb* adaptational phenotypes in sequential environments during the course of primary infection (based upon published transcriptomic studies).

Together, the transcriptomic studies of *M. tb* tell a story of *M. tb* adaptation from expectoration in sputum to transmission to infection and dissemination to EP sites where bacteria survive without eliciting granuloma formation (Figure 3). *M. tb* in sputum is an inert bacterium in terms of replication, respiration, and virulence exiting an infectious host, which remains so until it encounters cells within a recipient host alveolus. Phagocytosis by an AM will result in efforts to counter the stresses associated with this cell in order to survive and be carried to the interstitium and/or to the circulation. Alternatively, uptake by an AEC will result in an opportunity to activate and replicate dramatically as well as become more virulent and invasive allowing the bacteria to penetrate the alveolar barrier. Once in the blood circulation, the bacteria will remain active and invasive to establish infection in non-phagocytic cells at multiple EP sites. Once a non-alveolar, non-hostile niche is reached, *M. tb* lies quietly till the opportune conditions arise. In the blood, the bacteria may also be capable of sensing the immune status of the host (e.g., HIV status), wherein the active, virulent, and invasive adaptations are enhanced. This observation is consistent with the epidemiology associating HIV-co-infection with increased EPTB (Shafer et al., 1991; Onorato and Mccray, 1992; Yang et al., 2004; Golden and Vikram, 2005; Naing et al., 2013).

## Establishment of Other Bacterial Infections

To better understand the establishment of *M. tb* infection, we can examine mechanisms used by other bacterial pathogens that have to cross a mucosal barrier to establish infection. The molecular mechanisms for adherence, invasion, and dissemination employed by other pathogens, e.g., *Streptococci, Staphylococci, Listeria, Candida* (and many more), that also translocate across mucosal barriers to establish infection have been investigated more thoroughly (Jedrzejas, 2001; Ferry et al., 2005; Bonazzi et al., 2009; Nobbs et al., 2009; Chen et al., 2010; Jensch et al., 2010; Krishnan and Narayana, 2011; Vengadesan and Narayana, 2011). The common principle that has emerged is that these pathogens use repertoires of adhesins, toxins and extracellular enzymes to establish infection. Adhesins are cell-surface or secreted proteins expressed by pathogens to attach and invade the epithelial and/or EC of the mucosal barriers by binding to receptors on their cell-membranes or to ECM proteins, e.g., Lm, fibronectin, collagens 1–4, fibrinogen, elastin, thrombospondin, vitronectin, E-cadherin, plasminogen, etc. Toxins participate by causing damage to cells to expose the underlying ECM for bacterial adhesins and increase permeabilization of the mucosal barrier for bacterial (and inflammatory cell) traversal (Jedrzejas, 2001; Ferry et al., 2005). Extracellular enzymes contribute by acting as adhesins, and by causing cell-membrane changes (Pancholi and Chhatwal, 2003). Thus, *S. pneumoniae* uses, cbpA (choline binding protein A; adhesin), pspA (pneumococcal surface protein A; adhesin), and Ply (pneumolysin; toxin) for infection and dissemination from the lungs (Jedrzejas, 2001; Sanchez et al., 2011). *L. monocytogenes* expresses several adhesins (FbpA, ActA, InlA, and InlB) that promote attachment and invasion of the intestinal barrier (Suárez et al., 2001; Dramsi et al., 2004; Bierne et al., 2007) and Listeriolysin O (LLO) which is a toxin that impairs the integrity of the intestinal barrier (Richter et al., 2009). Similarly, *S. aureus* uses FnBP (Fn binding protein) for adhesion to the respiratory epithelium, Spa (*S. aureus* protein A) to activate inflammatory responses, and α-hemolysin to damage the alveolar barrier to promote hematogenous dissemination (Ferry et al., 2005; Soong et al., 2011). Extracellular enzymes such as the Streptococcal Surface Enolase (SE) and glyceradehyde-3-phosphate degydrogenase (GAPDH) have evolved to also function as adhesins in some pathogens (Li et al., 2015). The McaP of *Moraxella catarrhalis* is a lipase/adhesin (Timpe et al., 2003); the Ssp of *Staphylococcus saprophytycus* is a surface-associated lipase that contributes to virulence (Sakinc et al., 2005, Szabados et al., 2013). The Als family of adhesins in *Candida albicans* are multifunctional family of proteins (Hoyer and Cota, 2016). Adhesins, toxins, and enzymes also modulate inflammatory responses to support establishment of infection; thus, the Vn-binding extracellular adherence protein (Eap) of *S. aureus* inhibits neutrophil recruitment in mice (Chavakis et al., 2002) as does the SLS (Streptolysin S) of *S. pyogenes* (Lin et al., 2009) and β-hemolysin of *S. aureus* (Tajima et al., 2009). Modifications of the inflammatory responses could enhance and optimize the window of time required by the pathogen to avoid/reduce elimination by other innate mechanisms like killing by neutrophils.

## Adhesins of *M. tb*

As in other bacteria, in addition to cytotoxins, adhesins play a role in the establishment of *M. tb* infection. Rv0475 (HBHA) is a well-characterized adhesin of *M. tb* that binds to HSPG in the cell membrane of AEC and in the BM underlying AECs (Menozzi et al., 1996). *M. tb* Δ*hbha* is attenuated (~50% reduction compared to wild type) for adherence and invasion of AEC, but not of macrophages, and for dissemination from the lungs to spleen in mice (Pethe et al., 2001). Transcripts for *hbha* are upregulated during infection of AEC *but not* during infection of macrophages (Delogu et al., 2006) or RPE cells (Abhishek et al., 2018). Thus, HBHA contributes to direct dissemination of *M. tb*, independent of macrophages, and specifically from the lungs. Another *M. tb* adhesin which is CNS-specific is, Rv0931c (PknD) (Be et al., 2012). PknD is a Lm-binding adhesin that contributes to *M. tb* attachment and invasion of human brain microvascular EC but not to AEC or macrophages or non-CNS EC (Be et al., 2012). *pknD* is not differentially expressed in AEC or macrophages or RPE (Schnappinger et al., 2003; Fontán et al., 2008; Ryndak et al., 2015; Abhishek et al., 2018).

Besides functioning as a cytotoxin, ESAT-6 is also an adhesin that binds to Lm and cell-membranes of AEC (Kinhikar et al., 2010). Since *in vivo*, Lm is primarily concentrated at the basolateral surface of AEC and is a major component of the BM, ESAT-6 could contribute to the anchoring of intra-AEC *M. tb* onto the basolateral Lm-expressing BM and cause damage to the cells and the BM by pore formation, thus facilitating macrophage-independent *M. tb* dissemination via the alveolar wall. This is similar to the Mip adhesin/toxin of *Legionella* which binds collagens (i, ii, iii, iv, v, vi), lyses lung epithelial cells, and damages the BM to achieve dissemination (Wagner et al., 2007).

Unlike in other mycobacteria, Rv1837c (Malate synthase; MS, GlcB) of *M. tb* is expressed extracellularly, binds Lm, and serves as an adhesin that contributes to the attachment of *M. tb* to AEC (Kinhikar et al., 2006). The *M. tb* MS has a C-terminal 15 amino acid extended sequence that is absent in MS of *M. avium, M. leprae* or *M. smegmatis*. A peptide spanning this C-terminal region of *M. tb* MS shows binding to Lm, fibronectin, and AEC (Kinhikar et al., 2006). Like HBHA and ESAT-6, transcripts for MS are upregulated during *M. tb* replication in AEC (Ryndak et al., 2015). None of these adhesins are upregulated by *M. tb* in either the THP-1, mBMM, or RPE (Schnappinger et al., 2003; Fontán et al., 2008; Abhishek et al., 2018).

As for other bacteria, evidence for synergy between adhesins and toxins in infection/dissemination is also available for *M. tb*. In the complete *in vitro* bilayer model of the alveolar barrier where AEC and EC are separated by a BM, *M. bovis* BCG Δ*hbha*, which lacks both ESAT-6 (adhesin/toxin for AEC) and HBHA (adhesin for AEC), is even more attenuated than the single *esat-6* deficient mutants (an additional ~50%) for migration across the *in vitro* alveolar barrier (Ryndak et al., 2016).

## Potential Mechanisms for *M. tb* Uptake by AEC

Besides the adhesin:ECM interaction, evidence for AEC uptake of *M. tb* by receptor-mediated macropinocytosis involving actin-polymerization and signal transduction is now described (Scordo et al., 2016). While this mechanism is certainly less efficient than phagocytosis by AM, the far higher prevalence of AEC in the alveolus coupled to a highly permissive intracellular environment could have a profoundly significant effect during primary infection. Recently, Syndecan 4 (Sdc4) in AEC membranes has been demonstrated to be a receptor for HBHA (Zimmermann et al., 2016). The extracellular domain of Sdc4 contains heparan sulfate chains consistent with previous studies demonstrating HBHA binding to AEC depends on host cell surface heparan sulfate chains (Pethe et al., 2000). Consistent with the ~50% decrease in translocation of BCG Δ*hbha* across an *in vitro* model alveolar barrier (Ryndak et al., 2016), Sdc4 knock-out AEC are impaired for *M. bovis* BCG uptake by about 50% (Zimmermann et al., 2016).

Bacterial uptake by non-phagocytic cells such as epithelial cells, EC, and fibroblasts can also occur by bacterial targeting of lipid rafts on the host cell membrane. Lipid rafts are isolated, tightly packed regions of eukaryotic cell membranes which are enriched with cholesterol and glycosphingolipids, and may localize certain cell signaling molecules/receptors (Pike, 2003). Bacteria, such as *Pseudomonas aeruginosa, Chlamydia* spp., *Shigella flexneri, Salmonella typhimurium, E. coli* K1, and *Coxiella burnetii*, target lipid rafts in host cell membranes which concentrate/localize receptors and/or key host signaling molecules to facilitate entry (Lafont et al., 2002, Zaas et al., 2005; Lim et al., 2010; Toledo and Benach, 2015; Loh et al., 2017; Samanta et al., 2017). *M. tb* has been shown to induce lipid raft aggregation in the membranes of AEC, and this capability is associated with its internalization (Fine-Coulson et al., 2012). Thus, *in vitro M. tb* infection of type 2 AEC triggered the formation of lipid raft aggregates in the cell membranes, and at 24 h p.i. ~50% *M. tb* was observed to co-localize with lipid raft markers. Furthermore, disruption of lipid rafts by pretreating the AEC with Filipin significantly reduced the numbers of intracellular bacteria suggesting a role for lipid raft targeting and aggregation in *M. tb* internalization by type 2 AEC. Whether it is *M. tb* adhesin:lipid raft interactions that promote uptake by AEC (or other non-phagocytic cells), either directly or through co-localized receptors, remains to be investigated; however, adhesins of other bacterial pathogens have been shown to mediate their uptake by non-phagocytic cells through lipid raft-dependent mechanisms including *Chlamydia pneumoniae* (Fechtner et al., 2016), *Streptococcus pneumonia* (Yamaguchi et al., 2013), and Shiga-toxigenic *E. coli* (Rogers et al., 2012).

Bacterial pore-forming toxins can also engage host cell lipid rafts (Toledo and Benach, 2015). Cholera toxin activity and internalization into epithelial cells are inhibited when the cells are pretreated with Filipin, thus indicating lipid raft involvement (Orlandi and Fishman, 1998). Furthermore, the pore-forming toxin LLO, triggers aggregation of lipid rafts in macrophage membranes (Gekara and Weiss, 2004; Gekara et al., 2005), and LLO pore-forming activity has been linked to *L. monocytogenes* uptake by host cells (Vadia et al., 2011). Culture filtrate proteins from *M. tb* H37Rv and *M. tb* HN878 induce significantly higher numbers of lipid raft aggregates on AEC compared to untreated cells (~30–40 aggregates per cell compared to ~5–10 aggregates per cell) and infection of AEC with *M. tb* pretreated with amikacin to prevent bacterial protein synthesis did not result in any increase in lipid raft aggregates (Fine-Coulson et al., 2012) indicating secreted factor(s) of *M. tb* contribute to the formation of lipid raft aggregates and possibly *M. tb* internalization as well by AEC.

## A Vaccine Strategy for Preventing *M. tb* Infection

The scenario described in this review suggests that likely targets for the prevention of *M. tb* infection would be the adhesins, toxin(s), and extracellular enzymes that promote primary infection and dissemination through their interactions with AEC. Thus, vaccination that leads to the attenuation of the interactions of HBHA with AEC, for example, has been demonstrated. Pre-incubation of wild type *M. tb* with anti-HBHA antibodies attenuates the dissemination of the intra-nasally administered bacteria from the lung to spleen indicating a non-macrophage and pro-AEC mechanism for dissemination during primary infection (Pethe et al., 2001). Similarly, mucosal immunization with HBHA reduces dissemination of *M. Bovis* BCG from the lungs in mice, and this reduction correlates with elicitation of anti-HBHA antibodies (Kohama et al., 2008). Antibodies against the CNS-specific adhesin, PknD, reduce bacterial invasion of human brain microvascular EC (Skerry et al., 2013), and guinea pigs vaccinated with PknD are protected against *M. tb* dissemination to the brain but not to pulmonary bacterial burden, and this protection correlated with the generation of PknD-specific antibodies (Skerry et al., 2013). PknD, therefore, is another illustration of adhesin:target specificity in *M. tb*. Also, antibodies against the Lm-binding domain of MS reduce *M. tb* adherence to AEC (Kinhikar et al., 2006). Interestingly, TB patients have high titers of antibodies to MS, but not to the Lm-binding site (unpublished data). *In vivo* studies of MS as a potential vaccine component have yet to be performed.

## Targeting Initial Adherence and Invasion as a Vaccine Strategy

Evidence for the substantial promise of anti-adhesin/toxin/enzyme antibody-based vaccines is becoming available (Raynes et al., 2018; Solanki et al., 2018). For example, a recent study demonstrated that the intraperitoneal co-administration into mice of two Enterotoxigenic *E. coli* (ETEC) multi-Ag fusions, one representing two mutated ETEC toxins and the other representing the epitopes of 7 important ETEC adhesins, induced antibody responses to both the toxins and adhesins, which neutralize bacterial enterotoxicity and adherence to the human intestinal cell line, Caco-2 (Duan et al., 2018). Antibodies targeted to these toxins were also found to be present in both the serum and colostrum of intra-muscularly immunized pigs (Nandre et al., 2018), and suckling pigs born to mothers immunized during pregnancy were passively protected from ETEC-induced diarrhea. Importantly, a human Phase 1 trial in which volunteers were orally administered bovine colostral IgG antibodies to the ETEC fimbrial tip adhesin CFA/1 pilin sub-unit CfaE (or placebo) and subsequently challenged with ETEC and monitored for diarrhea, demonstrated that passive vaccination with antibodies directed against an adhesin can yield significant, protective efficacy in humans (Savarino et al., 2017).

Targeting adhesin interactions with cells of the alveolar barrier has also shown potential as a vaccine strategy in the case of *Streptococcus pneumoniae* (Mizrachi Nebenzahl et al., 2016). *S. pneumoniae*, the cause of pneumococcal disease, is spread through the inhalation of contaminated aerosols from infected individuals. PtsA is a *S. pneumoniae* protein that has a cytosolic enzymatic function and a cell wall adhesin function (Mizrachi Nebenzahl et al., 2016). Pre-incubation of A549 cells with recombinant PtsA (rPtsA) and pre-incubation of bacteria with anti-rPtsA antiserum inhibited the adhesion of *S. pneumoniae* to these cells. Similarly, pre-incubation of the bacteria with host cell protein peptides determined to bind PtsA inhibited bacterial colonization of lungs in mice. Importantly, immunization of susceptible mice with rPtsA protected the animals against intravenous lethal dose challenge with *S. pneumoniae*.

The mechanisms of these vaccine strategies are to disrupt the initial adherence or barrier-penetrating steps critical for the establishment of the bacterial infection. Here, we have highlighted the adhesin, HBHA, and the adhesin/lysin, ESAT-6, as vaccine targets to prevent *M. tb* penetration of the alveolar barrier; however, other *M. tb* proteins with adhesin or lytic properties may also be involved, e.g., possibly MS and the ESAT-6-like proteins whose encoding genes were upregulated in AEC. Also, bacteria:host cell adhesion is described as a multi-step process, with increasing specificities of interaction (Krachler and Orth, 2013). It is conceivable, therefore, to devise an anti-*M. tb*-infection vaccine based on the generation of neutralizing antibody responses against *combinations* of *M. tb* adhesins/toxins and/or their functional regions that prevent attachment/invasion of the cells in the lung that support replication, dissemination, and active disease, i.e., the AEC. Such a vaccination strategy could succeed in decreasing the overwhelming reservoir of LTBI.

## Concluding Remarks

TB remains a major cause of morbidity and mortality in a large proportion of our world. Our understanding of the host/pathogen factors and interactions that lead to dissemination and establishment of *M. tb* primary infection, however, is limited. These enigmatic steps in *M. tb* infection, combined with our incomplete knowledge of the true correlates of protection against *M. tb* infection in humans, have hindered our ability to develop strategies to prevent infection.

Once *M. tb* infection is established, many challenges remain to obtain accurate diagnosis and proper treatment. Notably, TB infection, after established, behaves differently in different individuals, can elude diagnosis and, if drug resistant, confound treatment. For example, the observable pathologies can depend on the immune competence of the infected individual, e.g., HIV-coinfected individuals have a higher propensity for EP/disseminated TB disease; however, lung lesions identifiable through chest X-ray may not be present and the TST may read as negative due to an impaired immune system. These issues present significant obstacles in identifying and controlling TB. Regardless of the manifestation in the infected host, for essentially all infected individuals, *M. tb* was acquired by inhaling contaminated aerosol droplets expelled from a person with active pulmonary TB thus bringing the common critical interaction back to the alveolus. Indirect support for the role of AEC is derived from studies of *M. tb* strains infecting individuals over a 5 year period in Arkansas, with patient demographics taken into consideration, which found that individuals infected with strains of the Beijing/W Lineage were 3-times more likely to have EP involvement even after controlling for patient-associated risk factors for EPTB (Kong et al., 2007). Interestingly, isolates of the highly successful *M. tb* strains Beijing and F15/LAM4/KZN adhere to and invade A549 cells 5 times more efficiently than other unique isolates of *M. tb* (Ashiru et al., 2010). There is likely a highly dynamic interplay between the consequences of *M. tb* encounters with the AM and AEC during primary infection. Together, these observations suggest that adaptation to the intra-AEC environment drives a more invasive and disseminative phenotype in *M. tb*.

Targeting of adhesins and toxins involved in the primary establishment of infection as a vaccine strategy would involve the humoral immune response. Whether it is antibodies that neutralize/attenuate adhesion/invasion of *M. tb* to AEC that contribute to the prevention of infection in about half of individuals with frequent and close contact with infectious TB patients remains to be investigated. Interestingly, while ESAT-6 is considered to be a promising vaccine component, the vaccine formulations, strategy, and evaluation has been designed to exploit its ability to elicit strong IFN-γ responses (van Dissel et al., 2010; Kwon et al., 2018). HBHA has been shown to induce a cytolytic CD4+ T cell subset in persons with LTBI but not active TB, suggesting a role for HBHA in the control of *M. tb* infection (Aerts et al., 2019), and both HBHA-induced IFN-γ responses and anti-HBHA IgA were found to be elevated in household contacts and community controls compared to untreated TB patients in TB-endemic Addis-Ababa, Ethiopia, suggesting HBHA can confer both protective cell-mediated and humoral immune responses (Belay et al., 2016). While the exact mechanisms of most vaccines are unknown, several successful vaccines have been designed to use the humoral immune response as their mode of protection, e.g., polio, tetanus, diphtheria and pertussis, hepatitis A, and MMRV vaccines. Like BCG, the live, attenuated varicella zoster vaccine relies on the CMI response as its correlate of protection (Siegrist, 2013); however, varicella zoster live has the drawback of waning efficacy (Levin et al., 2008). The newly available, Shingrix vaccine, a non-live recombinant Ag vaccine against varicella zoster, which, in addition to CMI, elicits a strong humoral response, has a more persistent efficacy than the varicella zoster live vaccine (Syed, 2018). TB generates a fine balance between humoral and CMI responses in its host, where the initial humoral responses wane as the CMI responses emerge (David, 1990). By the time a TB patient is identified, the CMI response dominates. Recent studies have reported that there are distinct differences between the humoral profiles of latently infected individuals and active TB patients that go beyond the role as mere biomarkers of disease state and indicate antibody function in controlling *M. tb* infection (Lu et al., 2016). Thus, antibodies from persons with LTBI were shown to be associated with increased antibody-mediated phagocytosis and cytotoxicity, increased Fc binding to the activating receptor Fcγ-RIIIa, and glycosylation structures that distinguish them from antibodies from active TB patients. Furthermore, the effect of treating primary human monocyte-derived macrophages infected with *M. tb* with pooled antibodies from each group (active or latent) revealed increased *M. tb*:lysosome co-localization, increased inflammasome activation, and decreased intracellular bacterial survival in the macrophages treated with LTBI-derived antibodies compared to those treated with active TB-associated antibodies. Therefore, antibodies can play a role in the control of *M. tb* infection, and we postulate that enhanced and targeted humoral immune responses to the critical mechanisms of primary infection, such as the *M. tb* interactions with AEC, could be exploited as a robust and novel vaccine strategy to prevent TB.

In this review, we have discussed an emerging aspect of the *M. tb*:host interaction that may contribute significantly to primary infection with *M. tb*; that interaction is with the AEC. The findings described in this review are consistent with a scenario involving AEC that is independent of macrophages and explains how *M. tb* replication, dissemination, and establishment of infection can outpace the CMI response. This scenario does not preclude a role for the AM in *M. tb* infection, but opens a whole, new way of understanding primary infection with *M. tb* that should be investigated in order to develop new strategies for preventing *M. tb* infection. Interrupting this interaction could prove crucial to averting the downstream obstacles/factors that drive TB infection, latent or active.

Based on the cumulative studies highlighted here, we suggest an explanation of the “Black Box” events of early *M. tb* infection during which the few, inhaled *M. tb* outpace the generation of adaptive immune responses to replicate and establish systemic infection. Upon entry they may invade AEC, rapidly replicate in this safe niche, and spread to neighboring cells, simultaneously fortifying themselves with the armamentarium required to migrate across the alveolar wall, spread via the circulation to various organs, and seed non-phagocytic cells, where they lie dormant, till the opportunity to reactivate arises. Therefore, we propose that the *M. tb*:AEC encounter may be critical to the establishment of primary infection in a naïve host and needs the focus of the scientific community to develop strategies for the *prevention* of *M. tb* infection.

## Author Contributions

SL: concept and critical revision. MR: writing and final approval. MR and SL: agreement to be accountable.

### Conflict of Interest Statement

The authors declare that the research was conducted in the absence of any commercial or financial relationships that could be construed as a potential conflict of interest.
